# Chondrogenesis and osteogenesis are one continuous developmental and lineage defined biological process

**DOI:** 10.1038/s41598-017-10048-z

**Published:** 2017-08-30

**Authors:** Yan Jing, Junjun Jing, Ling Ye, Xiaohua Liu, Stephen E. Harris, Robert J. Hinton, Jian Q. Feng

**Affiliations:** 1 Department of Orthodontics, Texas A&M University College of Dentistry, Dallas, TX 75246 USA; 2 Department of Biomedical Sciences, Texas A&M University College of Dentistry, Dallas, TX 75246 USA; 3 Department of Periodontics, University of Texas Health Science Center at San Antonio, San Antonio, TX 78229 USA; 4 Present Address: State Key Laboratory of Oral diseases, West China Hospital of Stomatology, Sichuan University, Chengdu, 610041 China; 5 Present Address: Department of Dental Research, Naval Post-Graduate Dental School, Navy Medicine Professional Development Center Walter Reed National Military Medical Center; Postgraduate Dental College Uniformed Services, University of the Health Sciences, 8955 Wood Road Bethesda, MD, 20889 USA

## Abstract

Although chondrogenesis and osteogenesis are considered as two separate processes during endochondral bone formation after birth, recent studies have demonstrated the direct cell transformation from chondrocytes into bone cells in postnatal bone growth. Here we use cell lineage tracing and multiple *in vivo* approaches to study the role of *Bmpr1a* in endochondrogenesis. Our data showed profound changes in skeletal shape, size and structure when *Bmpr1a* was deleted using *Aggrecan-Cre* ^ERT2^ in early cartilage cells with a one-time tamoxifen injection. We observed the absence of lineage progression of chondrocyte-derived bone cells to form osteoblasts and osteocytes in metaphyses. Furthermore, we demonstrated the key contribution of growth plate chondrocytes and articular chondrocytes, not only for long bone growth, but also for bone remodeling. In contrast, deleting *Bmpr1a* in early osteoblasts with *3.6 Col 1-Cre* had little impact on skeletal shape and size except for a sharp increase in osteoblasts and osteocytes, leading to a profound increase in bone volume. We conclude that chondrogenesis and osteogenesis are one continuous developmental and lineage-defined biological process, in which *Bmpr1a* signaling in chondrocytes is necessary for the formation of a pool or niche of osteoprogenitors that then contributes in a major way to overall bone formation and growth.

## Introduction

During fetal development, endochondral bone formation begins at a primary ossification center in the calcified cartilage. The formation of secondary ossification centers from the epiphysis begins after birth. Rapid postnatal longitudinal bone growth depends on the growth plate (GP) and articular cartilage. Endochondral bone formation, which accounts for over 80% of the skeleton volume, was thought to begin with cell death in hypertrophic chondrocytes, followed by the invasion of vascular associated osteoblast progenitor cells from the underlying bone marrow that erode the calcified cartilage and initiate angiogenesis, and then by bone marrow and periosteum-derived cells that deposit new bone^1, 2^. In other words, chondrogenesis (providing a template and then giving way to osteogenesis) was viewed as a closely-linked but a separate biological process from osteogenesis during endochondral bone formation. However, it is well documented that defects in cartilage will dramatically change the shape and size of the skeleton. How can the inherited message be transmitted from chondrocytes, which are supposed to be “removed” before bone formation, to bone cells? Importantly, the mammalian articular cartilage and rodent’s growth plate remain active during their lifespan, although their biological significance during adult-life span is unclear.

The cell fate of hypertrophic chondrocytes has been an issue of debate for decades^3^. A number of investigators have made a strong case for the ability of hypertrophic chondrocytes to transform into bone cells^4, 5^. In fact, very recent reports have shown that a great proportion of the chondrocytes directly transform into bone cells in the murine metaphysis and diaphysis^6–9^. Moreover, when chondrocyte-derived osteoprogenitor cells were isolated from the rodent GP, they differentiated into bone cells *in vitro* ^10^. Using cell lineage tracing methodologies, we showed that hypertrophic chondrocytes (HCs) are transformed into osteoblasts (Obs) and osteocytes (Ocys) in the mandibular condyle^11–13^. However, it is currently unclear how this cell transformation is regulated at the gene level.

Bone morphogenetic proteins (BMPs) are vital not only to osteogenesis^14–16^ but to chondrogenesis. For example, the conditional knockout of *Bmp2* (cKO) in chondrocytes leads to severe defects in chondrogenesis, although *Bmp4* cKO exhibits no apparent defects^17^. Embryonic studies also showed that the deletion of *Bmpr1a* or *Bmpr1b* in chondrocytes produced no apparent defects, but double-mutant mice developed a severe chondrodysplasia phenotype, suggesting redundancy but a functionally vital role for BMP signaling in endochondral bone formation^18, 19^. Interestingly, postnatal *Bmpr1a* cKO mice made by crossing to *Aggrecan*-*Cre* ^ERT2^ (*Acan*, a gene highly expressed in all chondrocytes) developed striking defects in cartilage and bones in both the limbs^20^ and mandibular condyle^21^, indicating a more critical role of BMPs after birth. However, deletions of *Bmpr1a* in early osteoblasts had little impact on the overall skeletal shape and length except for changes in the bone volume and bone thickness^15, 16, 22, 23^. Thus, it is critical to clarify the regulatory roles of *Bmpr1a* in the cell transformation from chondrocytes into bone cells using cell lineage tracing techniques.

In this study, we attempted to test the hypothesis that chondrogenesis and osteogenesis are linked into one continuous developmental and lineage defined biological process, using multiple animal models combined with the confocal imaging technique. Our data showed that the direct transformation and lineage progression of chondrocytes into osteoblasts and osteocytes occurs in both the growth plate and the articular subchondral bone during bone growth and remodeling, supporting the biological significance of chondrocytes over the entire mouse skeletal lifespan. We also demonstrated that deleting *Bmpr1a* in early chondrocytes leads to severe defects in the skeleton with complete lack of metaphyses, delayed and malformed epiphyses, and thinned cortical bone, implying the potential role of chondrocytes in periosteal bone expansion. However, removing *Bmpr1a* in early osteoblasts results in no major change in the overall bone shape but an increase in osteoblast differentiation and mineralization, leading to a dramatic increase in bone volume. Our data support a new theory that chondrogenesis (phase one) and osteogenesis (phase two) are one continuous biological process during endochondral bone formation and remodeling, in which *Bmpr1a* signaling in chondrocytes is necessary for the formation of a pool or niche of osteoprogenitors that then contributes in a major way to overall bone formation and growth.

## Results

### Deleting *Bmpr1a* in chondrocytes leads to malformed epiphyses and absence of metaphyses

Here we propose that chondrogenesis and osteogenesis are essentially one continuous biological process, such that the disruption of chondrogenesis necessarily impairs osteogenesis. To test this hypothesis, we first deleted *Bmpr1a*, an essential molecule required for both embryonic and postnatal chondrogenesis^18–20^, and activated *Rosa* tomato in chondrocytes using the *Acan-Cre* ^ERT2^ at postnatal day 3 (P3, one-time tamoxifen injection). These null mice displayed short and malformed skeletons (Fig. S1), confirming our previous findings that *Bmpr1a* is required for postnatal skeleton formation^20^. Furthermore, H&E-toluidine blue staining showed a nearly complete lack of hypertrophic chondrocytes in the cKO growth plate and trabecular bone at metaphysis 7 days after the Cre event was activated (Fig. S2).

**Figure 1:**
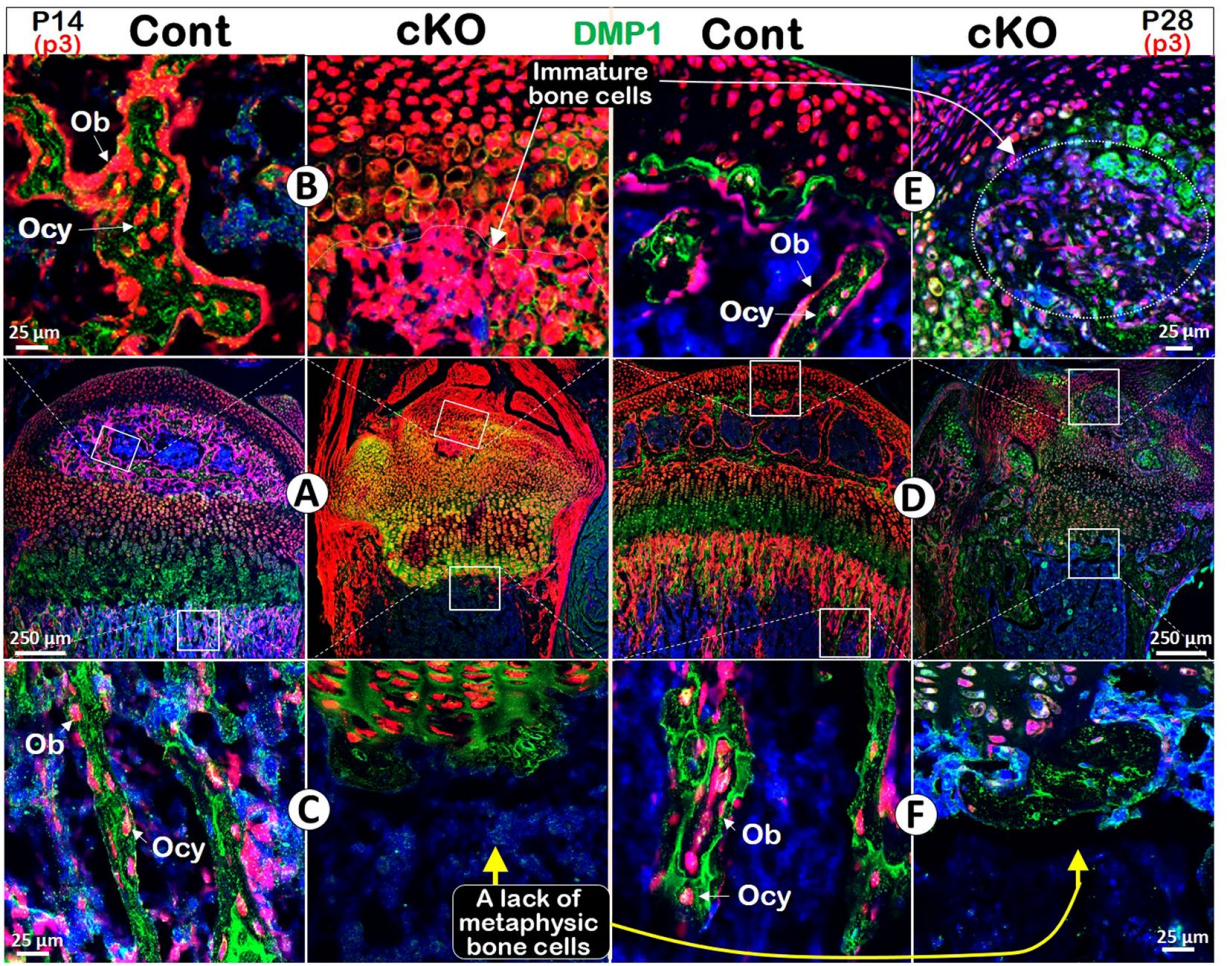
Distinct roles of Bmpr1a in epiphyseal and metaphyseal bone formation. (**A**–**C**) The control P14 confocal image (red, tomato; green, DMP1 IHC) revealed Tomato^+^ Ocys in the 2^nd^ ossification center with Tomato^+^ Obs on the bone surface. In the cKO epiphysis, there was no secondary ossification center but numerous red immature bone-like cells (**B**). In the control metaphysis, the Tomato^+^ bone cells filled the bone column under the growth plate. In the cKO, there was essentially no metaphysis except for a few green Ocys that originated from non-chondrocyte progenitors (**C**). (**D–F**) The merged P28 image showed a well-formed epiphysis, in which Tomato^+^ Ocy cells were buried in the secondary ossification center. In the cKO epiphysis, there was no articular cartilage with few mature Ocys (**E**). In the control metaphysis, the Tomato^+^ bone cells filled the bone column. In the cKO, there was no metaphysis (**F**).

To define the role of *Bmpr1a* in regulating the cell transformation, cell lineage tracing with *Acan-Cre* ^ERT2^ combined with immunohistochemical (IHC) staining was performed at P14 and P28, after Cre was activated at P3 by tamoxifen. DMP1 IHC results in the control showed that there were numerous Tomato^+^ chondrocyte-derived osteoblasts (Obs) on the bone surface with no DMP1 signal and osteocytes (Ocys) in the matrices that expressed DMP1 at P14 (11 days after the Cre event was activated). In the *Bmpr1a* cKO, almost all Tomato^+^ cells in the secondary ossification center were Ob-like, expressing little DMP1 (Fig. 1A,B). In the control metaphysis, Tomato^+^ Obs and Ocys accounted for large portion of bone cells, whereas in the cKO metaphysis, there were essentially neither Tomato^+^ Obs or Ocys nor bone tissue (Fig. 1A,C). This finding suggests that 1) a subset of bone cells (osteoblast/osteocytes) are of chondrogenic origin in the secondary ossification center, but not from the migrating bone cells as commonly believed; 2) chondrogenesis and a component of osteogenesis are linked biological processes; and 3) BMPR1A signaling is required for progression of a major pool of chondrocytes to mature osteoblasts and osteocytes in both epiphyses and metaphysis.

By P28 (25 days after the *Cre* event was activated in chondrocytes), the articular cartilage layer and subchondral bone were well formed in the control, in which the Ocys expressed DMP1. In the cKO, there were no detectable mature bone cells (Fig. 1D,E). In the cKO metaphysis, there was little trabecular bone and Tomato^+^ Obs or Ocys, pointing to an arrest of bone formation (Fig. 1D,F). Furthermore, Col II IHC staining confirmed the impact of the deletion of *Bmpr1a* in chondrocytes on the formation of both epiphysis and metaphysis at ages of P14 (Fig. S3A–C) and P28 (Fig. S3D–F), in which the cKO chondrocytes can transform into immature bone cells in the epiphysis. In the metaphysis, there was a more dramatic defect in the capacity of the *Aggrecan* ^+^ cells to transdifferentiate into mature Obs and Ocys.

### Removing Bmpr1a in chondrocytes induces a sharp reduction in bone cells and cortical thickness in the diaphysis

**Figure 2:**
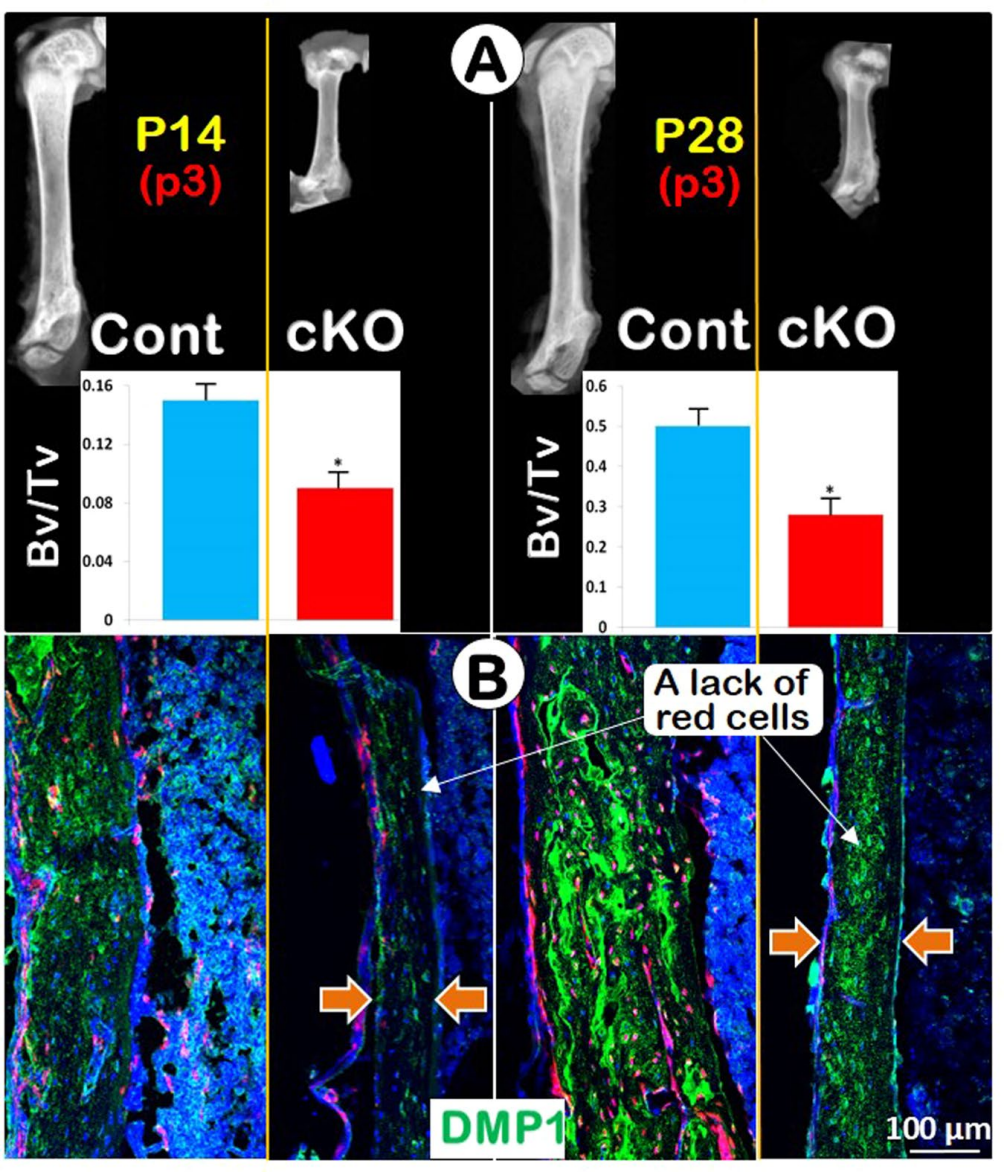
Bmpr1a is a regulator of cortical bone formation. (**A**) The x-ray images for P14 and P28 groups showed short thin-walled femurs from cKO mice. The quantitative μCT data (bars) showed a significant reduction of BT/TV in cKO diaphysis at both ages (n = 4; *P < 0.05). (**B**) DMP1 IHC staining at P14 and P28 confirmed that there were dramatic reductions of cortical bone volume and chondrocyte-derived osteocytes in the *Bmpr1a* cKO mice.

It has been reported that chondrocyte-derived bone cells directly contribute to cortical bone formation, either using the *Acan-Cre* ^ERT2^ line^8^ or the *Col10a1*-*Cre* line^10^. The mechanisms governing this regulation and process are unclear. Here, our X-rays showed a great reduction of cortical bone thickness in the diaphysis of femurs from *Bmpr1a* cKO mice at both P14 (Fig. 2A, *left panels*) and P28 (Fig. 2A, *right panels*). μCT data demonstrated a statistically significant difference of BV/TV between the cKO and the age-matched control mice in diaphysis (Fig. 2A, bars for both P14 and P28 stages). Subsequently, we showed a low expression in DMP1 IHC with few Tomato^+^ (the *Acan-Cre* ^ERT2^-*R26R* ^Tomato^) Ocys in the *Bmpr1a* cKO cortical bones at P14 and P28 (Fig. 2B). Furthermore, we compared the number of Tomato^+^ bone cells and cartilage matrix (using Col II IHC), and found a sharp decrease in red Tomato^+^ bone cell numbers and a low expression of type II collagen (green) in two *Bmpr1a* cKO age groups (Fig. S4). These data indicates *Aggrecan* ^+^ cells contribute to the formation of cortical bone under the regulation of *Bmpr1a*.

### Chondrocyte-derived bone cells contribute to late stage bone modeling

**Figure 3:**
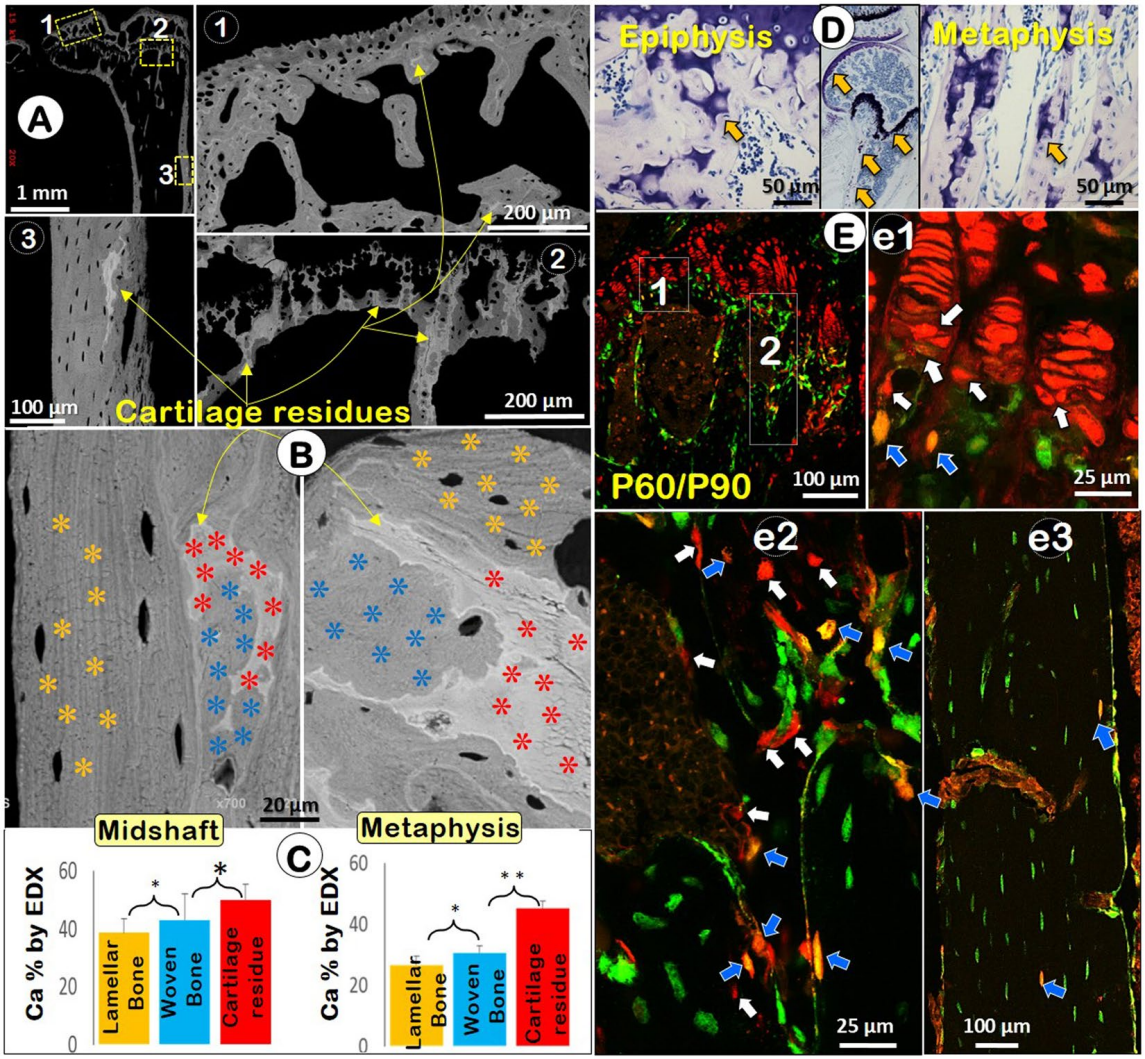
The EDX (Energy-dispersive X-ray spectroscopy) and cell lineage tracing data support the role of chondrocyte-derived bone cells in bone remodeling (3-month-old long bones). (**A**) The backscattered SEM image displayed numerous cartilage residues (brighter white) in the articular subchondral bone (a1), metaphysis (a2) and midshaft cortical bone (a3). (**B**) Three regions based on the morphological characters in the SEM images were selected for measurements of Ca% with 9 points picked from each area (yellow, laminar bone; light blue, woven bone; and red, cartilage residue). (**C**) The quantitative EDX data showed a significant difference in the Ca% among different mineralized areas (n = 4; *P < 0.05; **P < 0.01). (**D**) The toluidine blue image showed cartilage residues in the matrix and in the Ocy cytoplasm. (**E**) The overlapping confocal images revealed red chondrocytes (essentially all), and mixed bone cells (red, white arrows; yellow, blue arrows) in the borderline between hypertrophic chondrocytes and bone cells (e1), metaphysis (e2) and cortical bone (e3).

The rodent GP persists throughout the lifespan, although its function after growth is unclear. To evaluate the contribution of chondrocyte-derived bone cells to bone remodeling, we first noted numerous cartilage residues in the adult subchondral bone, metaphysis, and cortical bone in backscattered SEM images (Fig. 3A and a1–a3, in white color). Calculations of calcium (Ca) percent in the laminar bone, woven bone and cartilage residue in both the diaphysis and metaphysis were performed using the quantitative EDX analysis approach on the same SEM images in the Fig. 3A (Fig. 3B, stars). Surprisingly, the highest Ca level was detected in the cartilage residues followed by woven bone, and the lowest level in laminar bone. These differences were statistically significant (Fig. 3C). Next, we showed cartilage residues were present in bone from the epiphysis, metaphysis and diaphysis by the toluidine blue stain (Fig. 3D, arrows). To further examine the direct transformation of chondrocytes to bone cells in the adult mouse, we injected Tamoxifen into a 2-month-old compound mouse (containing *R26R* ^Tomato^; *2.3Col1a1*-GFP; *Acan-Cre* ^ERT2^) and harvested the bone samples at the age of 3 months. The confocal image displayed Tomato^+^ chondrocytes in the entire growth plate (i.e., remaining active) and a mixed bone cell population in the metaphysis (Fig. 3E and 3e 1–e2, red, yellow and green), and diaphysis (Fig. 3e 3), indicating a continuing role of chondrocyte-derived bone cells in rodent during the late modeling phase (also see Fig. S5).

### *Bmpr1a* is a key regulator of long bone remodeling in chondrocyte-derived bone cells

**Figure 4:**
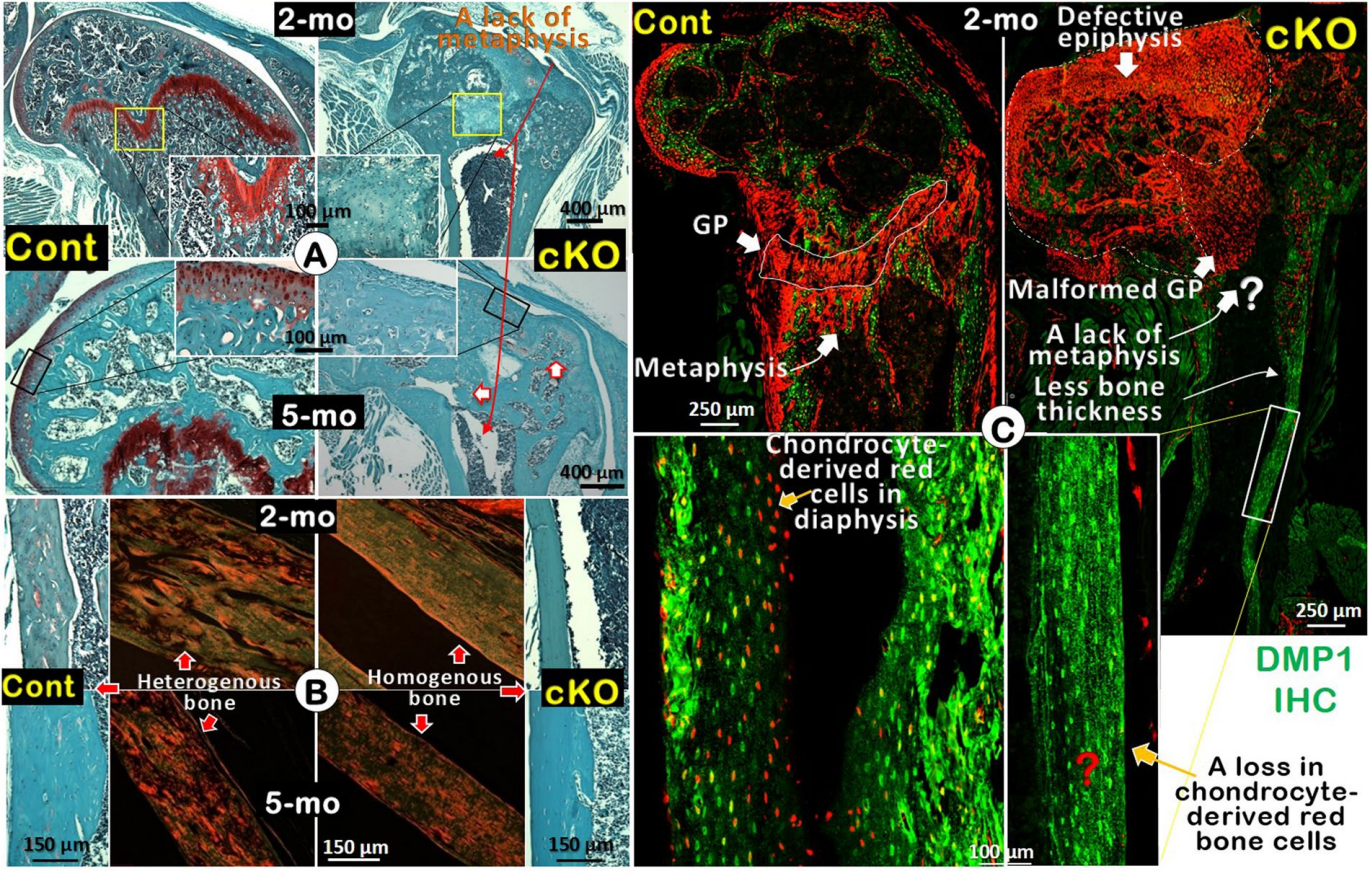
Removing *Bmpr1a* in chondrocytes results in severe defects in epiphysis, a lack of metaphysis, a sharp reduction in midshaft bone thickness, a change in bone matrices from heterogenous bone to homeogenous bone, and a lack of chondrocyte-derived bone cells in the diaphysis. (**A**) Safranin O staining for 2- (*upper*) and 5-month (*lower*) mice revealed a lack of mature cartilage in articular cartilage and GP and an absence of metaphysis. (**B**) In the midshaft, there were few cartilage residues, leading to a laminar-like bone in the diaphysis of cKO mice (*right*) by Safranin O stain. The polarized-light-microscope-images confirmed that the heterogenous cortical bone in the control (*middle left*) and homogenous laminar bone in the cKO (*middle right)* at both age groups. (**C**) DMP1 IHC (green color) showed Tomato^+^ mature chondrocytes in the articular cartilage and GP, and chondrocyte-derived bone cells in the midshaft bone in 2-month control mice (*lower left*). In contrast, neither mature chondrocytes in the articular cartilage and GP, nor Tomato^+^ bone cells in the metaphysis and midshaft bone, were displayed in the cKO mice. Partial loss of GP led to a direct connection between the epiphysis and midshaft (GP: growth plate).

To test the biological significance of chondrocyte-derived bone cells during remodeling, we analyzed *Bmpr1a* cKO mice at adult ages. At the age of 2 months, cartilage matrix was detected by Safranin O staining in control growth plate, but was absent in age-matched cKO growth plate (Fig. 4A, *upper panels*). We also noted little or no staining in the articular cartilage region in the cKO at this stage. By 5 months, Safranin O staining remained strong in articular cartilage and GP in control mice (indicating an active stage), whereas there was essentially no articular cartilage nor GP in the cKO mice (Fig. 4A, *lower panels*), supporting a critical role of Bmpr1a in maintaining active chondrogenesis in adult stage. The red cartilage residues detected by Safranin O staining showed the control cortical bone matrix to be a heterogeneous mixture of woven and laminar bone in appearance (Fig. 4B, *left panels*). In contrast, there were no cartilage residues nor chondrocyte-derived woven bone in cKO mice, resulting in the dominant appearance of laminar bone (Fig. 4B, *right panels*). The polarized light microscopic images further confirmed this finding, in which there was a mixed distribution pattern of woven and laminar bone in the control in contrast to a uniform distribution pattern in the cKO (Fig. 4B).

Importantly, the 2-month-old cell-lineage tracing data revealed numerous Tomato^+^ chondrocyte and bone cells in the articular cartilage, epiphysis, growth plate, metaphysis and diaphysis of control mice (Fig. 4C, *left*). In contrast, the cKO articular cartilage and GP displayed few mature chondrocytes, a lack of a metaphysis, and absent Tomato^+^ bone cells in the diaphysis (Fig. 4C, *right*).

### Eliminating *Bmpr1a* in non-chondrocyte-derived bone cells has no apparent effect on overall bone shape and size

**Figure 5:**
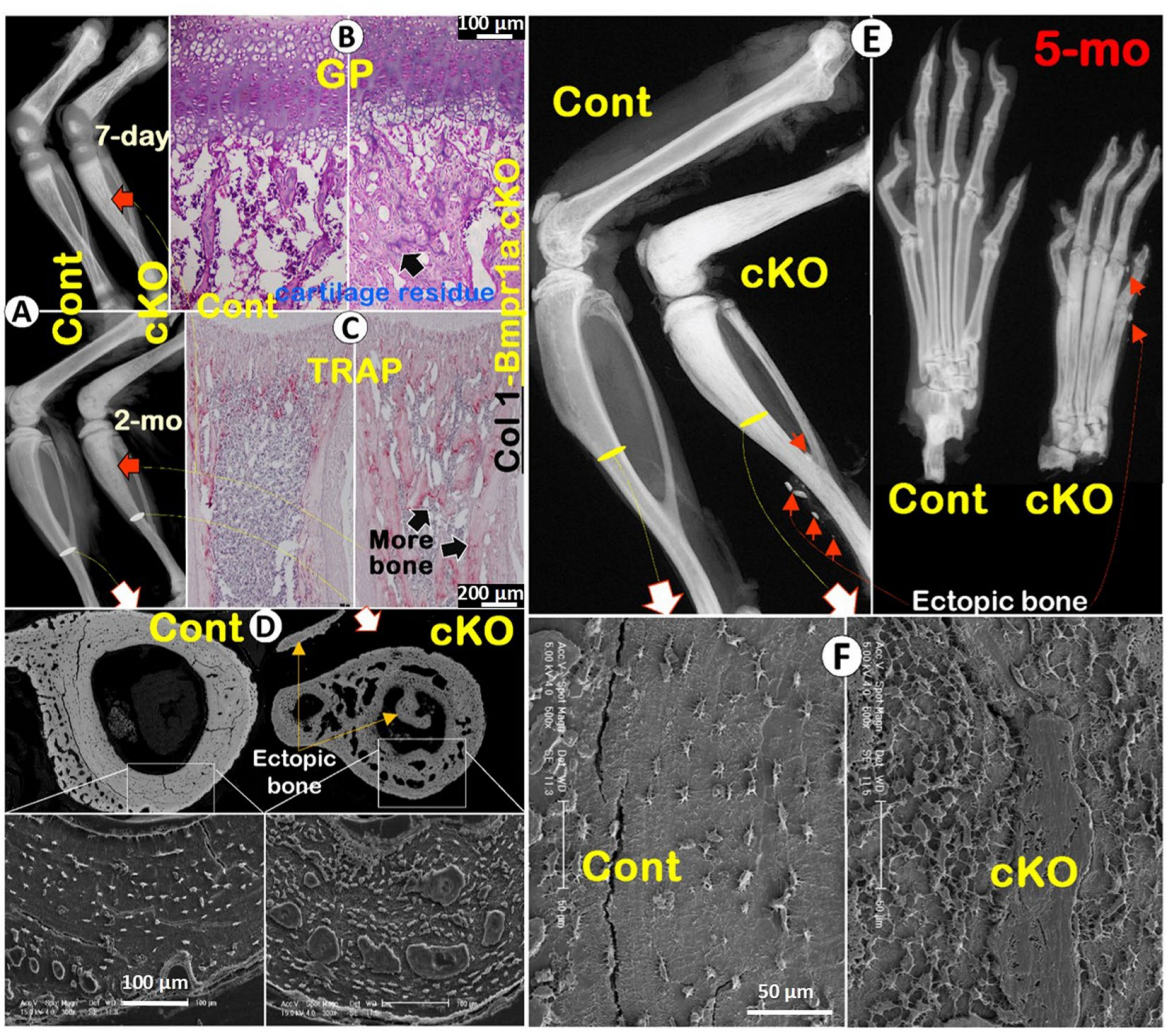
*Bmpr1a* in non-chondrocyte derived early osteoblasts negatively controls cell number and bone mass during bone development and remodeling. (**A**) The X-ray images showed no apparent changes in skeletal shape at the age of P7 (*upper panel*) and 2-months (*lower panel*) except for additional bone in the bone marrow after deleting *Bmpr1a* by *3.6 kb Col 1-Cre* (*right panels*). (**B**) H&E staining displayed no apparent difference in GP and cartilage residues in the cKO at the age of P7 (*right*). (**C**) The TRAP assay displayed a slightly lower osteoclast number/bone surface area but more trabecular bone in the P7 cKO (*right*). (**D**) The SEM images from 2-month-old mice exhibited bone cells and ectopic bone in the cKO (*right*). (**E**) The X-ray images from 5-month-old mice exhibited more severe ectopic bone formation in the cKO bone marrow, tendons and soft tissues (*right panels*, arrows). **(F**) The SEM images showed numerous bone cells, indicating a continuous role for *Bmpr1a* in control of bone cell number and bone mass (*right*).

One of the potential disadvantages of using the *Acan-Cre* ^ERT2^ line is that the Cre event was also observed in the periosteum and bone marrow progenitor cells^8^, indicating that some of the bone phenotypic changes may be due to a direct consequence of a loss of *Bmpr1a* in non-chondrocyte bone cells. To address this concern, we deleted *Bmpr1a* in non-chondrocyte-derived early osteoblasts using the *3.6 kb Col 1-Cre* separately^24^. These cKO mice showed more bone in the 7-day-old and 2-month-old long bones (Fig. 5A). The H&E stain (Fig. 5B) and TRAP stain (Fig. 5C) images demonstrated no apparent change in the growth plate and the cartilage residue distribution pattern in the bone matrix except for greater bone mass, and more bone cells plus ectopic bone formation in the cKO (Fig. 5D). By 5 months the cKO bone marrow was fully filled with bone mass in both the large and small bones (Fig. 5E). The acid-etched SEM images revealed enormous Ocys in the cKO cortical bone. As a result, removing *Bmpr1a* in non-chondrocyte-derived early osteoblasts leads to an increase in cell proliferation but a decrease in cell differentiation. The *Bmpr1a* cKO bone cells, which are immature, fail to form fully mineralized bone, leading to numerous non-mineralized foci in the bone (Fig. 5D lower panel, Fig. 5F). This finding agrees with other reports, in which *Bmpr1a* was deleted by the *3.2 Col 1-Cre* ^15, 16^, the *Osx-Cre* or the *Dmp1-Cre* ^23^.

## Discussion

Chondrogenesis has been traditionally viewed as a discrete process with little direct contribution to bone formation, except for its possible role in bone fracture repair. The current studies suggest 1) chondrogenesis (phase one) and osteogenesis (phase two) are one continuous biological process that is regulated by *Bmpr1a*; 2) the shape and size of the skeleton are mainly determined by chondrocyte-derived-bone cells that also contribute to bone remodeling; and 3) the growth plate and articular cartilage not only contribute to long bone growth, but also to bone remodeling via the transformation of chondrocytes into bone cells.

It has been well documented that chondrogenesis is crucial for determining skeletal shape and size^25^. Yet, there is a knowledge gap regarding how the inherited message is relayed from chondrocytes to bone cells, as hypertrophic chondrocytes have been long thought to die prior to bone formation. To solve this puzzle, we first studied the cell fate of the *Bmpr1a*-null chondrocytes (containing the *R26R* ^Tomato^ reporter as a cell tracing marker) combined with IHC for cartilage and bone markers during postnatal endochondral bone formation. Our data displayed no replacement of chondrocytes by migrating Obs in the secondary ossification center, in which all bone-like cells are Tomato^+^ (with few bone marker expressions) and derived from the *Bmpr1a* null HCs, indicating a continuous process of a subset of chondrocytes differentiating to osteoblasts and osteocytes (Fig. 1). Moreover, there is essentially no new bone formed in the metaphysis after removing *Bmpr1a* in chondrocytes, further supporting the notion that normal chondrogenesis contributes a major portion of chondrocytes to the pool of osteoprogenitors (Fig. 1). In contrast, deleting *Bmpr1a* in the classic osteogenic cells has no apparent effect on overall skeleton shape and size (Fig. 5). Together, these data argue strongly that it is the chondrocyte-derived bone cell that carries and transmits the crucial ‘memory’ (i.e., the inherited message) for defining the pattern, structure and growth of the skeleton postnatally. In addition, the above studies support the notion that this coupling of chondrogenesis and osteogenesis is regulated by *Bmpr1a* in *Aggrecan* ^+^ cells.

It has been known for decades that cartilage residues buried in bone matrices cannot be removed by surface osteoclasts, although there was little exploration of this “common knowledge” due in part to the impact of the old concept that chondrogenesis is a separate event from osteogenesis. In this study, we demonstrated disparities in Ca percent between laminar bone and woven bone using backscattered SEM quantitative EDX analysis (Fig. 3). It is possible that differences among these various sub-regions of bone may indirectly reflect the variant cell resources that produce the bone: e.g. non-chondrocyte-derived bone cells for laminar bone versus chondrocyte-derived bone cells for woven bone.

Currently, the only accepted role of chondrogenesis in bone remodeling is in bone fracture repair^26^, although cartilage matrices in growth plates and their residues remain in adult bones with no apparent linkage to its biological contribution to bone remodeling. To address this enigma, we first confirmed that chondrocytes in adult mice continue to secrete cartilage matrix in articular cartilage and growth plates, and that cartilage residues persist in the subchondral bone and in metaphyseal and diaphyseal bone. Using the cell lineage tracing technique, we also showed that young adult cartilage cells stay active (as they continuously express the tomato reporter), and the transformation of chondrocytes into bone cells persists (as reflected by the red and yellow bone cells in both metaphysis and diaphysis areas) (Figs 3 and 4). These data strongly support an active role of chondrocyte-derived bone cells in bone formation during the late modeling phase. Further, we deleted *Bmpr1a* in chondrocytes and showed a lack of mature cartilage cells in the adult GP and articular cartilage. As a result, the articular cartilage of *Bmpr1a* cKO mice is progressively replaced by fibroblast like tissue, and the GP of the *Bmpr1a* cKO mice was completely gone, resulting in a “canal” between epiphysis and metaphysis (Fig. 4). In addition, normal cartilage residues are not detectable in the diaphyseal cortical bone of the *Bmpr1a* cKO mice, in which the cortical bone is almost completely composed of laminar-like bone matrix (homogeneous), in contrast to the age-matched control, in which the cortical bone contains laminar and woven bone with numerous cartilage residues (heterogeneous) (Fig. 4). Thus, we propose that chondrocyte-derived bone cells continue to contribute to bone remodeling, rendering the bone heterogeneous.

The Col II^+^ chondrocytes in the cKO remained in early stages and failed to form mature hypertrophic chondrocytes in the *Bmpr1a*-null mice (Fig. S6A). We speculate that this defect is partially caused by dramatic reductions in the levels of SOX9 and Osterix (OSX) in *Bmpr1a* null mice, two critical transcriptional factors for chondrogenesis (Fig. S6B,C).

The demonstration that a one-time injection of tamoxifen to remove *Bmpr1a* in cartilage at P3 results in a lack of long bone growth postnatally supports the notion that the *Aggrecan* ^+^ progenitor/stem cell numbers in cartilage are limited. In other words, the stockpile of progenitor cells present in the growth plate in a lifetime are largely fixed, and cannot be augmented with more new progenitor cells after the growth is completed. This observation may explain why the rodent growth plate remains unfused during the animal’s lifetime but there is essentially little bone lengthening after maturity. However, we showed that there is a very small amount of Tomato^+^ chondrocytes, which then contribute to the normal bone replacement during late bone modeling and remodeling phase.

**Figure 6:**
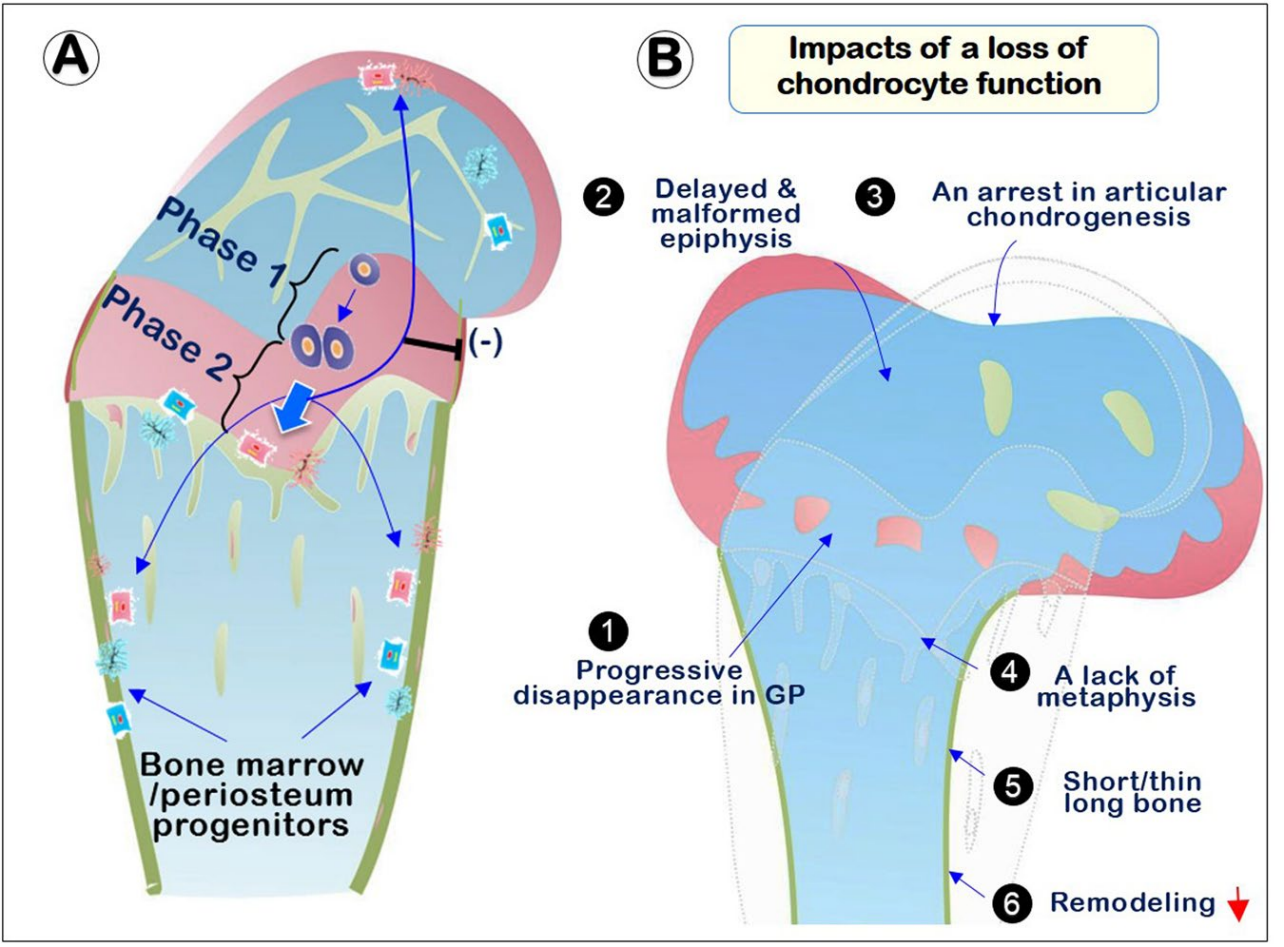
Working Hypothesis: the impact of chondrocyte-derived bone cells in endochondral bone formation and remodeling. We propose that one set of progenitor cells directly forms bone cells without going through the chondrogenic step, and a second set of progenitor cells forms chondrocytes first and then bone cells. (**A**) Two-phases but one continuous developmental and lineage defined biological process during endochondral bone formation and remodeling: chondrocyte maturation (phase one) occurs first. Many of these cells directly transform into bone cells (phase two). These transformed bone cells, which carry the original inherited message for determining the shape and size of the skeleton, contribute mainly to the formation of bone in epiphysis and metaphysis but also to bone formation in the diaphysis. Our data also support the continuous role of chondrocyte-derived bone cells in bone remodeling. *Bmpr1a* regulates endochondral bone formation and remodeling, but by different mechanisms. (**B**) The impact of chondrocyte-derived bone on the shape and size of the skeleton: the conditional deletion of *Bmpr1a* in chondrocytes leads to: 1) a progressive loss of structure and function in GP (growth plate); 2) a delayed and malformed epiphysis; 3) an arrest in articular chondrocyte/subchondral bone formation; 4) a lack of metaphysis; 5) short and thin long bone; and 6) a defect in long bone remodeling.

In summary (Fig. 6), this study combining multiple animal models and state of the art techniques presents convincing evidence that chondrocyte-derived osteogenesis is a major contributing program to bone formation during early postnatal endochondral formation and late stages of bone replacement and remodeling. These chondrocyte-derived osteoblasts and osteocytes are essential for bone formation, not only in the epiphysis and metaphysis, but also contribute to woven bone formation in the cortical bone of diaphysis. *Bmpr1a* in chondrocytes is a critical pathway for this transdifferentiation process. Finally, our data revealed the key contribution of growth plates and articular cartilage, not only for long bone growth, but for bone remodeling after the initial rapid bone growth. It is highly likely that it is the chondrocyte-derived bone cells that carry the inherited message defining the shape and size of the skeleton.

## Experimental Procedures

### Breeding transgenic mice

To generate triple mice for cell lineage studies of the fate of chondrocytes in endochondral bone formation, *Acan*-*Cre* ^ERT2^ ^27, 28^, *2.3Col1a1-GFP* mice^29^, and *R26R* ^Tomato^ (B6; 129S6-*Gt(ROSA)26Sor* ^*tm9(CAG-tdTomato)Hze*^/J, the Jackson Laboratory) were internally crossed three times. One-time tamoxifen i.p. injection was performed at postnatal P3 or 2 months (75 mg/kg body weight). To study the influence of deletion of *Bmpr1a* in chondrocytes on cell fate, we generated triple mice by internally crossing *Acan*-*Cre* ^ERT2^, *Bmpr1a* ^flox^ ^30^, and *R26R* ^Tomato^ three times. One-time tamoxifen i.p. injection was performed at P3 with animals euthanized at P14, P28, 2-months, 3-months or 5-months. To delete *Bmpr1a* in non-chondrocyte derived early osteoblasts, *Bmpr1a* ^flox^ mice were crossed to *3.6 Col 1-Cre* ^24^. One-time tamoxifen i.p. injection was performed at P3 with animals euthanized at different time intervals from P7, P14, 2-months or 5-months. All protocols were reviewed and approved by the Institutional Animal Care and Use Committee at Texas A&M University College of Dentistry, and all methods were performed in accordance with relevant guidelines and regulations. A total of 74 animals (4–6 animals a group), which ranged from one-week to five-months of age containing both sexes, were used in these experiments.

### Immunohistochemistry, Toluidine blue and Safranin O staining

For decalcified bone analysis, animals were fixed by using freshly prepared 4% paraformaldehyde in PBS (pH 7.4). Then samples were decalcified and embedded in paraffin as described previously^31^. Sections were cut at 4.5 µm thickness and were mounted on slides and dried. Slides were used for immunohistochemistry, Hematoxylin & Eosin (HE), Safranin O, Toluidine blue, and TRAP staining as formerly reported^32, 33^. The concentrations of the primary antibodies for the immunohistochemistry are: rabbit polyclonal anti-Col I (Abcam, 1:100); mouse anti-Col II monoclonal antibody (Santa Cruz Biotechnology, 1:50); rabbit polyclonal anti-Osterix (1:400, Abcam, Cambridge, England); rabbit polyclonal anti-Sox9 (1:100, Santa Cruz Biotechnology, Dallas, TX, USA); rabbit polyclonal anti-DMP1 (a gift from Dr. Chunlin Qin, TX A&M University College of Dentistry).

### Confocal microscope

Long bones were fixed in 4% paraformaldehyde and decalcified at 4 °C, followed by CryoJane frozen sectioning as previously described^34^. Fluorescent cell images were captured using a SP5 Leica confocal microscope made in Germany. All images were captured at light ranging from 488 (green)-561 (red) mm wavelengths. Multiple stacked images were taken at 200 Hz and 1024 × 1024 in dimension using 10x or 20x or 63x glycerol objective lenses. For both the control and null mice, a thickness of 8 µm was obtained with a pixel size of 0.1542 µm × 0.1542 µm × 0.49 µm^35^.

### Radiographs and micro-CT

Radiographs of bone samples dissected free of muscle were taken using a Faxitron model MX-20 Specimen Radiography System with a digital camera (Faxitron X-Ray Corp., Lincolnshire, IL, USA). Long bone µCT analysis was performed by Scanco µCT35 (µCT35; Scanco Medical, Bassersdorf, Switzerland). Serial tomographic imaging was done at an energy level of 55 kV and intensity of 145 µA. Cortical bone thickness data was obtained at the midshaft of the bone. Therefore, 100 cross section of slices above the midshaft of femur were analyzed at a threshold of 283. Bone volume vs total volume at the midshaft was calculated and used for comparison of the samples.

### Backscattered scanning electron microscopy (SEM), acid-etched SEM, and Energy-dispersive X-ray spectroscopy (EDX)

The freshly isolated hind limbs were fixed in 4% paraformaldehyde solution at room temperature for 48 hours. The tissue specimens were dehydrated in ascending concentrations of ethanol (from 70% to 100%), embedded in methyl-methacrylate (MMA, Buehler, Lake Bluff, IL)^36^. The MMA embedded samples were cut and the surface polished using 1 μm and 0.3 μm alumina alpha micropolish II solution (Buehler), followed by acid etching with 37% phosphoric acid for 2 to 10 seconds, 5% sodium hydrochloride for 5 minutes and then coated by gold (for acid-etched SEM imaging). For backscattered SEM and EDX, samples were coated by carbon after polish. Samples were scanned by a FEI/Philips XL30 field-emission environmental SEM (Hillsboro, OR, USA) as described previously^37^.

EDX is an analytical technique used for spectral analysis of individual elements such as calcium, oxygen and phosphate. The value of each element relies on an interaction of X-ray excitation from each sample. Capability to characterize each element is mainly due to its atomic structure displaying a unique set of peaks on its X-ray emission spectrum. The structural mineral content (calcium) observations and elemental energy-dispersive X-ray microanalysis were based on the method described previously^38^. Briefly, a total of 3 polished control bone blocks coated with carbon (from mice at 3-months of age) were imaged by back-scattered electrons and analyzed for the elemental composition of the minerals (calcium) present. Nine spots (~2 µm^2^) were picked up from three areas: cartilage residues, woven bone and laminar bone in metaphyses and diaphyses separately.

### Statistics

All data are reported as mean values ± SEM. The Kruskal–Wallis test was used to detect any significant differences among samples. The Mann–Whitney U test (post hoc test) was used to compare differences between the age-matched control and cKO group. Significance level is defined as follows for all analyses performed: *p < 0.05; **p < 0.01.

## Electronic supplementary material

Supplementary information
