# Supplementary material for: Chondrogenesis and osteogenesis are one continuous developmental and lineage defined biological process
Source: Sci Rep. 2017 Aug 30;7:10020. doi: 10.1038/s41598-017-10048-z (PMC5577112; doi:10.1038/s41598-017-10048-z)
Supplement: Supplementary file 1 — Supplementary information [file 41598_2017_10048_MOESM1_ESM.pdf]

Chondrogenesis and osteogenesis are one continuous developmental and lineage defined biological process

Yan Jing[1*], Junjun Jing[2], Ling Ye[2], Xiaohua Liu[2], Stephen E. Harris[3], Robert J. Hinton[2], Jian Q. Feng[2*]

SUPPLEMENTAL INFORMATION

Fig. S1. Removal of *Bmpr1a* in chondrocytes by crossing *Bmpr1a*^{flox} and *Acan-Cre*^{ERT2} with one time injection of tamoxifen at age of postnatal day 3 (P3) leads to a lack of bone growth plus a malformed skeleton.

(A) Representative radiographic images showed a cessation of long bone growth at both stages: 1-month and 5-months. Of note, there was a bony protrusion at the knee joint in the cKO mice.

(B) Alizarin red/Alcian blue staining of the control (cont, *left*) and the cKO whole skeleton (*right*) at age of one month is shown, in which the bone was retarded in the cKO epiphysis.

Fig. S2. *Bmpr1a* is essential for postnatal limb development.

Use of Toluidine blue/H&E staining shows a dramatic decrease of metaphyseal bone formation at P10 (7 days after tamoxifen injection) in the *Acan-Cre*^{ERT2}; *Bmpr1a* cKO mice.

Fig. S3. Cell lineage tracing shows distinct responses in different regions in *Bmpr1a* cKO mice: a lack of maturation in chondrocyte-derived bone cells in the epiphysis and a complete cessation of cell transformation in the metaphysis.

(A) The merged P14 confocal images, in which red bone cells were derived from chondrocytes and the green matrix was IHC staining using anti-Col II antibody, revealed red osteocyte (Ocy)-like cells in the secondary ossification center with red osteoblast (Ob) cells on the bone surface (*upper left*). In the cKO epiphysis, there were numerous red immature Ocy-like cells trapped in the HC lacunae (*upper right*, arrows), and few red Obs on the bone surface, indicating a critical role for *Bmpr1a* in regulating

bone cell maturation after cell transformation. In the control metaphysis, red bone cells filled the bone column under the growth plate (*lower left*). In the cKO, there were essentially no HC cells in the GP nor bone cells in the metaphysis (*lower right*), supporting the notion that *Bmpr1a* is essential for chondrocyte maturation in metaphysis.

(B) The merged P28 confocal image showed a well-formed epiphysis in which red bone cells were observed in subchondral bone with cartilage residues stained with Col II IHC (*upper left*). In the cKO epiphysis, there was a lot of articular cartilage stained with Col II but few mature bone cells and a lack of cartilage residues (*upper right*). In the control metaphysis, the red bone cells filled the bone column (*lower left*). In the cKO, there was no metaphyseal bone (*lower right*). Taken together, the above time-line data suggest that *Bmpr1a* is indispensable for control of chondrocyte-derived bone cell maturation.

Fig. S4. The cartilage residues are dramatically decreased in *Bmpr1a* cKO cortical bone in development stage.

Col II IHC staining showed that there were many red chondrocyte-derived bone cells in the cortical bone surrounding Col II$^+$ cartilage residues in the P14 and P28 control mice. The thickness of cortical bone, the number of red chondrocyte-derived bone cells and the strength of Col II IHC were all sharply reduced both age groups of cKO mice.

Fig. S5. Cartilage residues are present in the cortical bone of adult wild type mice but residues are largely undetectable in age-matched *Bmpr1a* cKO cortical bone.

(A) Backscattered SEM images obtained from the 2-month-old tibias showed cartilage residues in the control mouse (*left*, white color), whereas cartilage residues were not

detectable in the cKO mouse line: *Acan-Cre*^{ERT2}; *Bmpr1a*$^{flox/+}$; *R26R*^{Tomato} (tamoxifen/harvest time at P3/P60).

(B) Col II IHC revealed Col II signal (green) in the control tibia cortical bone (*left*) but not in *Bmpr1a* cKO (tamoxifen/harvest time at P3/P60).

Fig. S6. Removal of *Bmpr1a* by *Acan-Cre*^{ERT2} leads to reductions in SOX9 and OSX in the null chondrocytes

(A) Col II IHC signal in the *Bmpr1a* cKO matrix is similar to that in the control.

(B) SOX9 IHC showed a strong signal in GP (growth plate) chondrocyte nuclei in the control mice but very weak in the *Bmpr1a* cKO mice.

(C) OSX expression was present in the control but was largely undetectable in the *Bmpr1a* cKO mice.

Fig S1

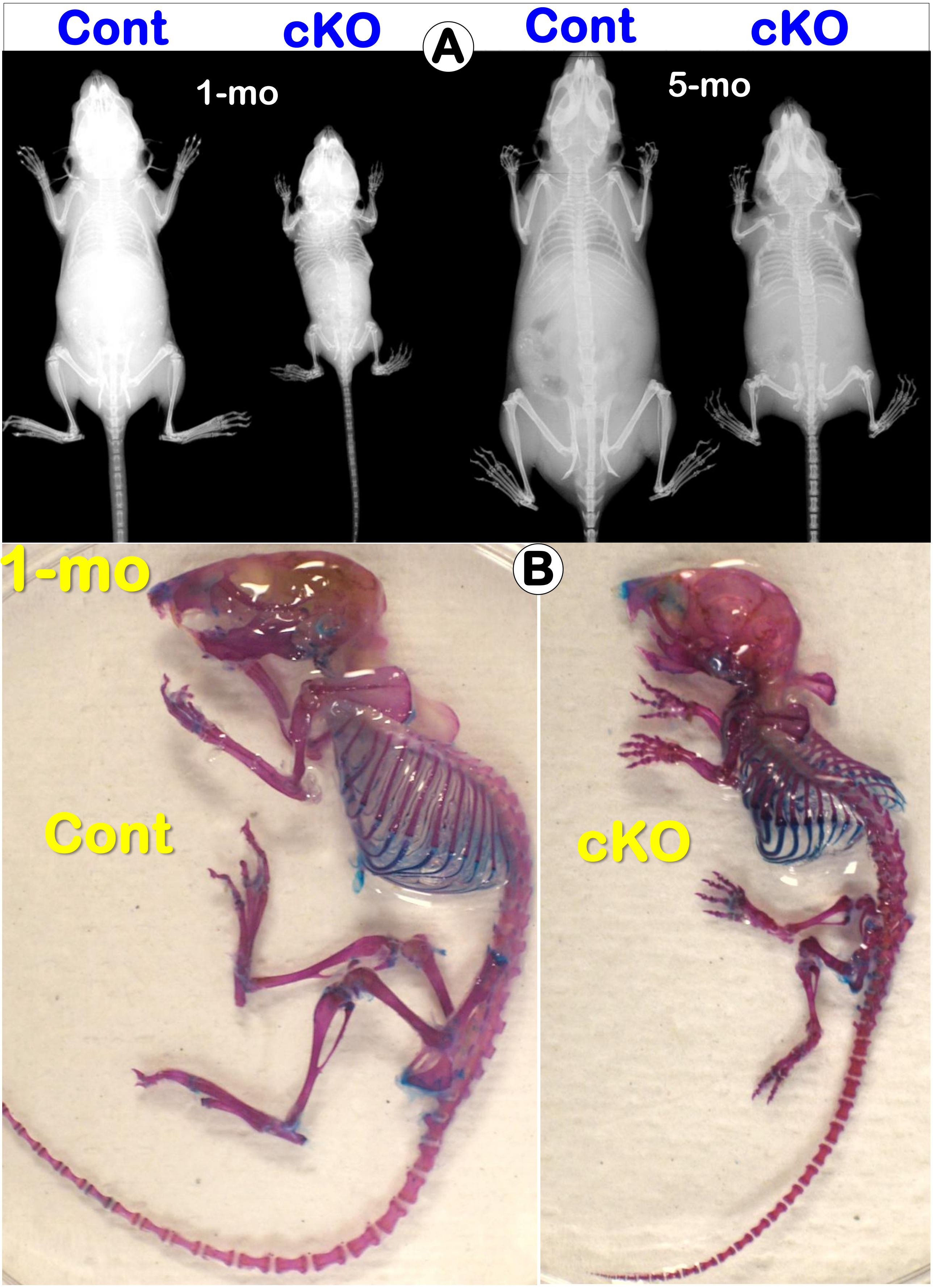

Fig S2

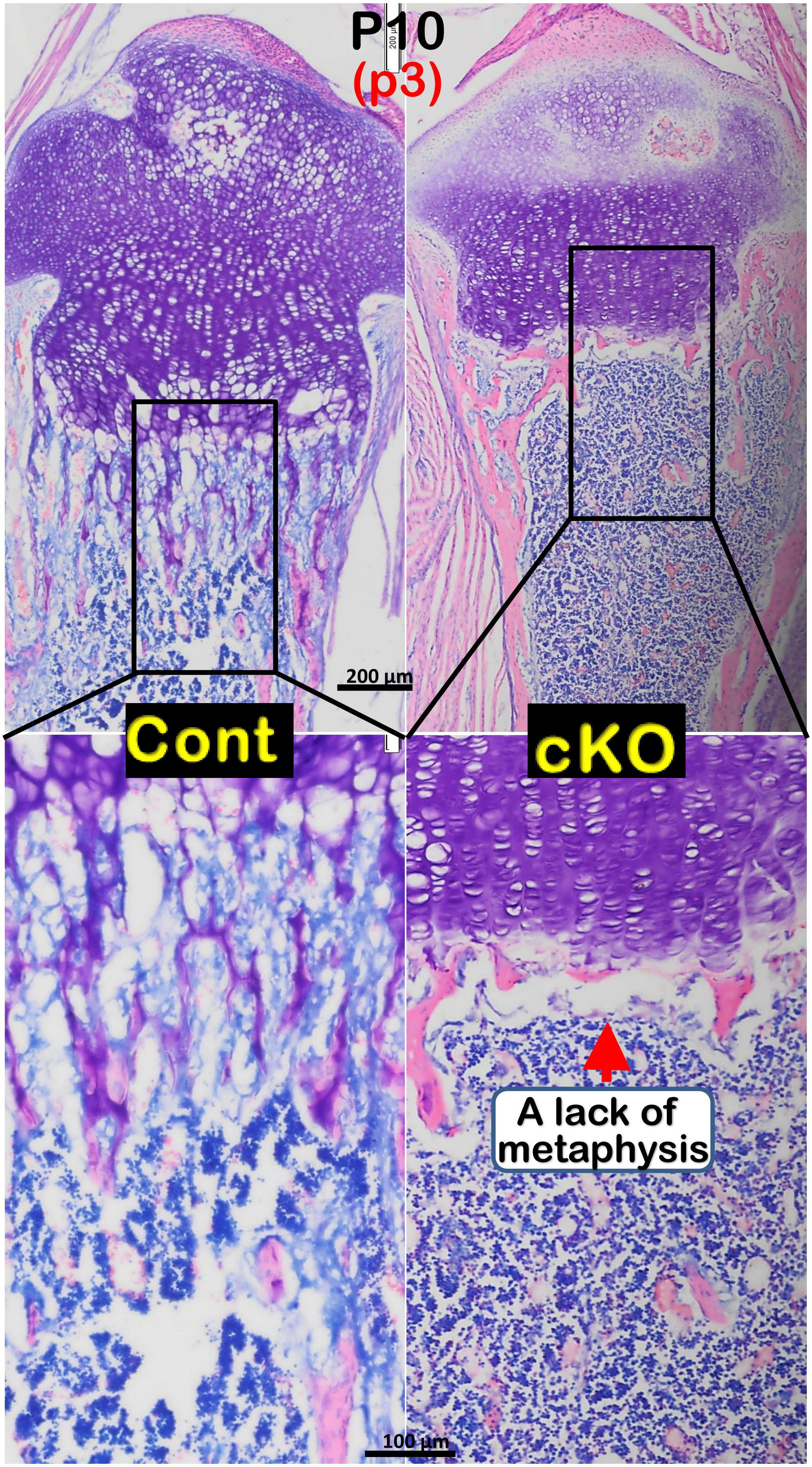

Fig S3

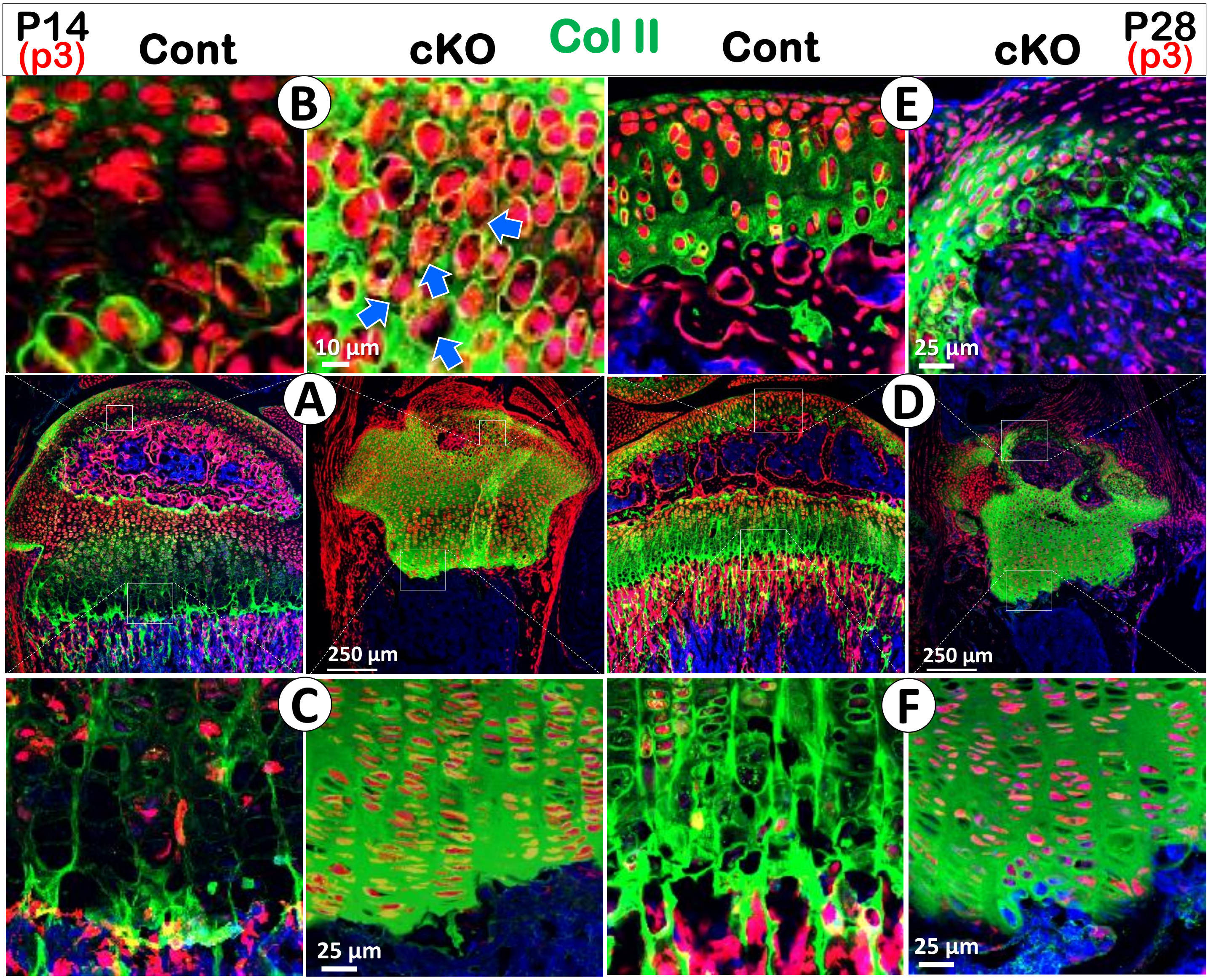
P14
(p3)
Cont
cKO
Col II
Cont
cKO
P28
(p3)
B
E
10 µm
25 µm
A
D
250 µm
250 µm
C
F
25 µm
25 µm

Fig S4

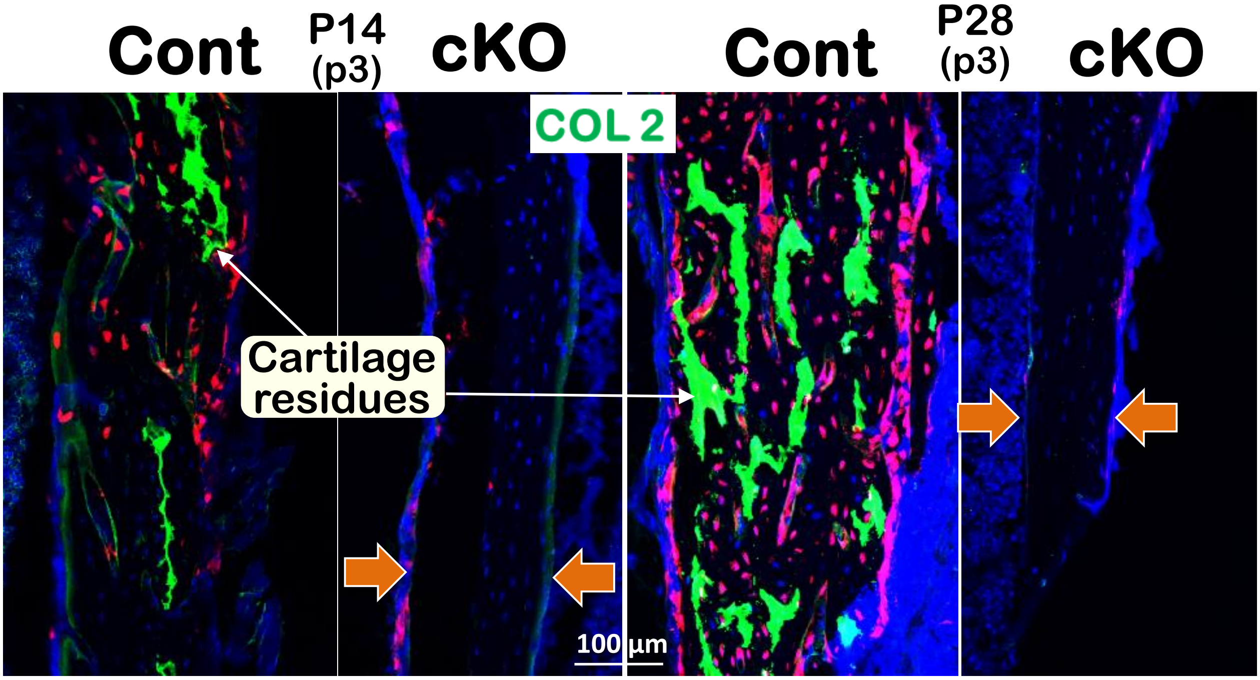
Cont
P14
(p3)
cKO
Cont
P28
(p3)
cKO
COL 2
Cartilage residues
100 µm

Fig S5

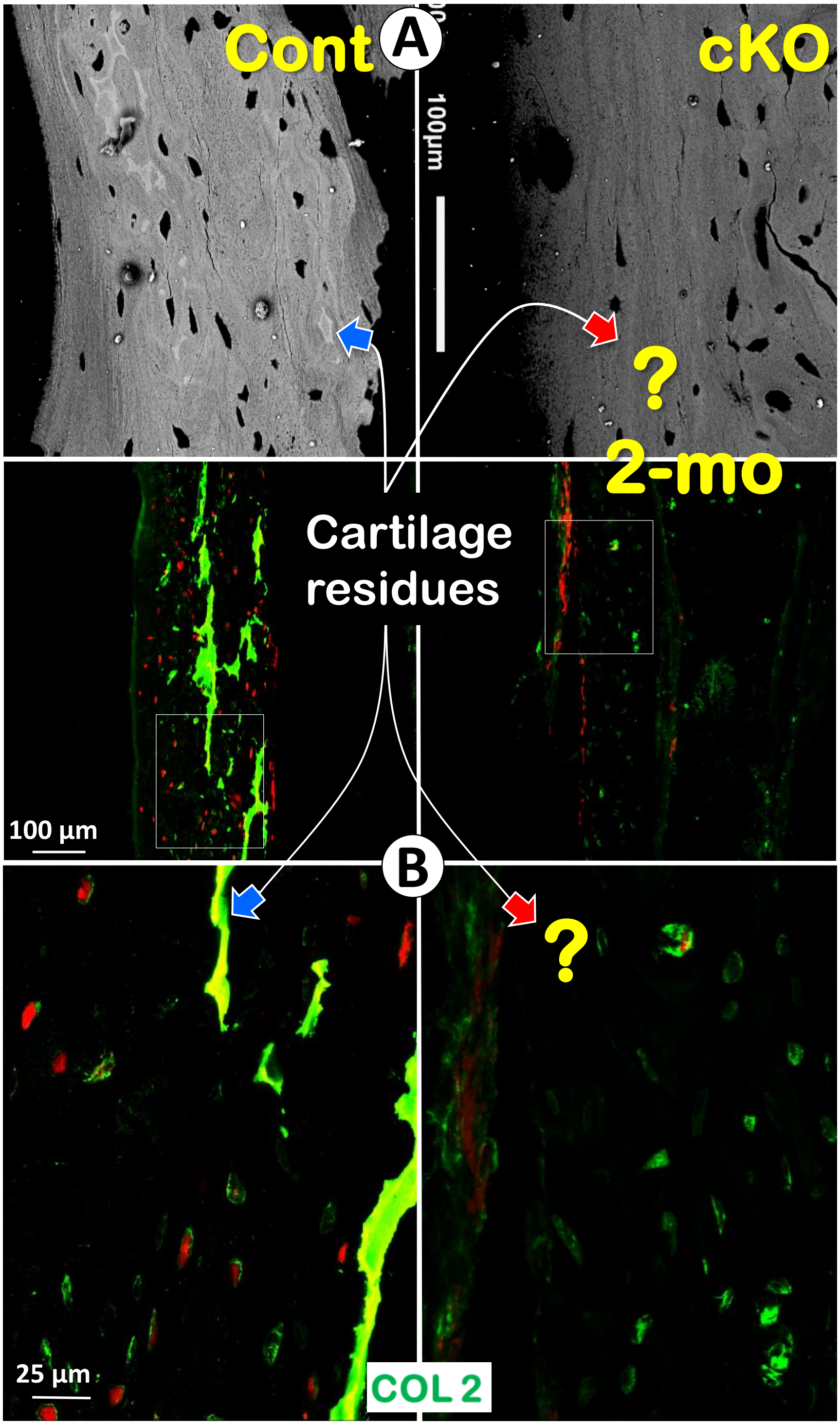

Fig S6

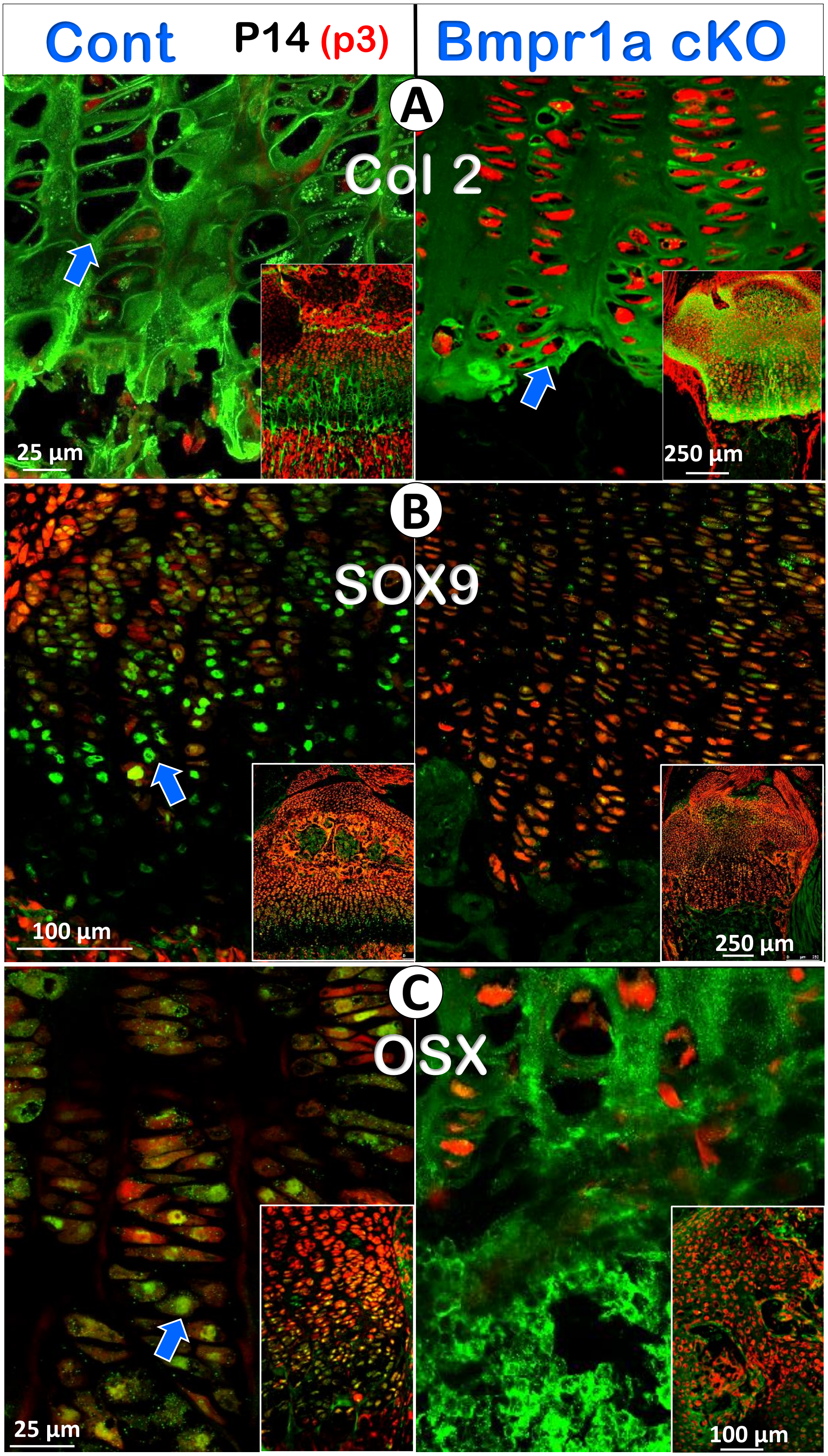

Cont
P14 (p3)
Bmpr1a cKO
A
Col 2
25 µm
250 µm
B
SOX9
100 µm
250 µm
C
OSX
25 µm
100 µm
